# Health Security inequalities in Non-EU European Countries: A Cross-National Comparative Assessment Using an Integrated MCDM-Machine Learning Approach

**DOI:** 10.12688/f1000research.163662.1

**Published:** 2025-04-24

**Authors:** Mijahed Nasser Aljober, Adel A. Nasser, Abed Saif Ahmed Alghawli, Amani A. K. Elsayed

**Affiliations:** 1 Department of Artiﬁcial Intelligence, Modern Specialized University, Sana'a, Yemen; 2 Department of Information Systems and Computer Science, Sa’adah University, Sa’adah, Yemen; 3 Department of Computer Science, College of Sciences and Humanities, Prince Sattam bin Abdulaziz University, Al Kharj, Riyadh Province, 16700, Saudi Arabia

**Keywords:** health security, Non-EU European Countries, K-means, clustering, CoCoSo, Entropy weighting method, MCDM

## Abstract

**Objectives:**

In an increasingly interconnected world, the effectiveness of health security (HeS) is pivotal in shaping informed health policies and enhancing public health outcomes. This study aims to analyses HeS in 27 non-EU European countries, identifying key priorities and trends, benchmarking against African and Eastern Mediterranean regions (EMR), and ranking and clustering health security performance to inform targeted interventions.

**Methods:**

Utilizing 2019, 2021, and aggregated 2017–2021 data from six Global Health Security Index indicators, this study applied an integrated Entropy-CoCoSo-K-means framework. The Entropy method was employed to identify health security (HeS) priorities and trends in Non-EU countries, enabling cross-regional comparisons with African and EMR regions to highlight priority shifts and disparities. The Entropy-CoCoSo (Combined Compromise Solution) model generated dynamic rankings, while K-means clustering categorized countries into five risk clusters (high to dangerous). This integration facilitated cross-national dynamic rankings and cluster analyses, informing targeted interventions across Non-EU countries.

**Results:**

Entropy analysis reveals that detection and reporting emerged as the most critical indicator (weight: 0.388), reflecting disparities in surveillance. The risk environment remains minimally influential (0.067), highlighting consistent vulnerabilities to external threats. Compliance with norms shows a sharp rise (0.091 → 0.123), indicating emerging regulatory gaps or uneven adherence to health standards post-2019. Cross-regional comparisons highlighted a focus on detection and reporting in non-EU countries versus an emphasis on prevention in Africa and healthcare infrastructure prioritization in the EMR. Ranking and clustering revealed stark disparities: Armenia, Norway, and the UK consistently ranked “High,” In contrast, Andorra, Monaco, San Marino, and Tajikistan (Cluster 5: “Dangerous”) exhibited systemic weaknesses.

**Conclusion:**

This study underscores the need for tailored policies to address non-EU Europe’s evolving HeS challenges. Harmonizing surveillance systems, scaling preventive measures, and bridging compliance gaps are critical. Regional collaboration and resource reallocation to low-performing nations are essential to mitigate disparities.

## 1. Introduction

Global health security has become a central focus in international policymaking, especially after the COVID-19 crisis exposed systemic weaknesses in healthcare infrastructures across nations. The pandemic underscored gaps in preparedness, response mechanisms, and equity in access to care. According to the World Health Organization (WHO), health security encompasses measures to minimize the danger and impact of acute public health events that endanger people’s health across geographical regions and international boundaries. ^1^ Similarly, the Global Health Security Agenda (GHSA) emphasizes the capability to mitigate, identify, and manage infectious disease risks, irrespective of their origin—natural, accidental, or intentional. ^2^

Health security is a multifaceted concept that goes beyond disease control to encompass a comprehensive framework that integrates preparedness, early detection, rapid response, and effective recovery from health emergencies. To achieve this, effective mitigation of public health risks is crucial, which in turn requires resilient healthcare infrastructures, multi-sector partnerships, and alignment with global frameworks like the International Health Regulations (IHR). Building on this foundation, a coordinated global effort is necessary to ensure that everyone is protected equally, and this effort should focus on three key areas: strengthening resilience, enhancing surveillance, and implementing strategic interventions to fortify health infrastructures. ^3^

Recent scholarly analyses have highlighted the persistent systemic barriers to robust health security governance in non-EU European countries, underscoring the need for urgent, coordinated interventions to address the multifaceted challenges that these countries face. The complex interplay of political instability, ^4^ economic disparities, ^5^ and climate change impacts ^6^ in non-EU European countries compounds vulnerabilities in their public health systems. The COVID-19 pandemic has exposed significant systemic weaknesses in global health security, particularly in these countries. Fragmented healthcare systems, inadequate disease surveillance, economic and social inequalities, limited financial and logistical capabilities, and inequitable resource distribution were key barriers that undermined effective pandemic responses. ^7– 9^ These deficiencies not only hindered the capacity to respond to health threats but also exacerbated inequalities in health outcomes.

Furthermore, the competitive dynamics among European nations, encompassing both EU and non-EU members, significantly impact European security cooperation, including health security. As noted by Simón (2017), European countries frequently emphasize their individual strengths while minimizing their weaknesses, which can undermine collective security initiatives. ^10^ This competitive environment may lead to fragmented approaches to health security, thereby constraining cooperation and mutual support. This fragmentation is particularly evident in the challenges encountered during joint procurements and negotiations for medicines and vaccines. ^11^

The challenges facing non-EU European countries are compounded by a dynamic geopolitical landscape characterized by rising nationalism and populism, which often obstruct collaborative health policies and long-term planning. ^12, 13^ This fragmentation not only hampers political cooperation but also exacerbates systemic health security issues within these nations. As a result, the inadequacies in governance manifest in underfunded infrastructure and widening health inequities.

In particular, studies have highlighted critical gaps in healthcare capacity across these regions, including shortages of medical resources, workforce deficits, and insufficient disease surveillance systems, especially in low- and middle-income settings. ^14, 15^ Moreover, the post-communist transitions in Eastern Europe have further deepened these disparities, as reliance on out-of-pocket payments has limited accessibility to healthcare services, adding another layer of complexity to the existing challenges. ^16^ Essential medical services, such as intensive care, neuroimaging, and clinical laboratories, continue to be under-resourced, exacerbating the crisis, along with a significant shortage of emergency-trained healthcare workers. ^17^

The COVID-19 pandemic has also exposed systemic weaknesses in routine immunization. Aguinaga-Ontoso et al. (2025) report significant declines in DTP3 vaccination coverage (2012-2023) across non-EU nations, risking reversals in progress against vaccine-preventable diseases and disproportionately harming marginalized and rural populations. ^18^ Economic instability, as highlighted by Thomson et al. (2022), ^19^ undermines resilience in countries dependent on volatile financing models during crises.

Together, these factors illustrate a concerning cycle in which political and economic instability, inequities in resource distribution, and disparities in services directly affect health outcomes, underscoring the urgent need for coherent strategies that address governance and health system deficiencies in non-EU European countries.

Notably, the GHSI, ^20^ a valuable tool for evaluating health security capabilities across nations, has faced criticism for its methodological shortcomings. Recent critiques by Refs. 21– 23 have highlighted these limitations, underscoring the need for more nuanced and comprehensive assessments of health security capacities. Specifically, critics argue that the GHSI’s rigid scoring system assigns equal importance to all evaluation criteria, overlooking regional variations in risks, resources, and health priorities, which can lead to distorted preparedness evaluations and misallocated resources. Furthermore, the index’s lack of mechanisms for multi-level geospatial analysis, including sub-regional weighting, ranking, and clustering, prevents nuanced spatial analysis, making it difficult to identify disparities between neighbouring countries or design interventions for overlapping crises. Additionally, the GHSI’s static framework cannot track changes over time, failing to adapt to shifting conditions, such as those observed during the COVID-19 pandemic. These shortcomings collectively highlight the urgency of revising the index to better reflect dynamic, context-specific realities, ensuring it becomes a more precise and actionable tool for shaping effective global health strategies.

To address the limitations of the GHSI, recent studies ^21– 23^ have proposed a novel approach that integrates multi-criteria decision-making (MCDM) methods with machine learning clustering. By synthesizing findings from a range of studies ^24– 37^ these researchers advocate for the development of a dynamic, context-sensitive evaluation frameworks that can capture the complexities of health security.

For instance, Study ^22^ systematically analyzed health security disparities in the Eastern Mediterranean Region (EMR) using a hybrid entropy-VIKOR-K-means model. The authors integrated six Global Health Security Index (GHSI) indicators with multicriteria decision-making (MCDM) techniques. First, entropy weighting—a data-driven method that assigns objective weights to criteria based on their inherent variability—was applied to prioritize health security dimensions. Next, the VIKOR method ranked countries by balancing overall performance against minimizing regret values, while K-means clustering categorized them into five stratified groups (high and low) using Euclidean distance metrics. Their analysis revealed stark disparities: high-income Gulf Cooperation Council (GCC) states like Qatar ranked highest due to robust detection systems, whereas conflict-affected non-GCC nations (e.g., Yemen, Somalia) exhibited systemic collapse in healthcare infrastructure, underscoring income as a critical determinant of preparedness.

Furthermore, Study ^23^ introduced a D-CRITIC-CoCoSo-K-means hybrid model for Western Asia (WA). The D-CRITIC method—a criteria weighting technique that accounts for both contrast intensity (data divergence) and correlation conflicts—replaced entropy weighting to reduce redundancy in GHSI indicators. The CoCoSo (Combined Compromise Solution) method then ranked countries by aggregating multiple compromise scores, while K-means clustering partitioned them into three distinct groups using D-CRITIC-CoCoSo composite scores.

Extending these methodologies to Africa, Study ^23^ employed an entropy-TOPSIS-K-means framework to evaluate 47 nations. Here, entropy weighting assigned criteria weights, while TOPSIS (Technique for Order Preference by Similarity to Ideal Solution) ranked countries by proximity to an ideal health security profile. K-means clustering revealed spatial inequities among African nations. All studies utilized publicly available GHSI datasets (2017–2021) and ensured reproducibility through Excel-based tools. Collectively, these hybrid models demonstrate how integrating MCDM with clustering can dynamically weight criteria, track trends, and geospatially prioritize regions—enabling policymakers to tailor interventions to local vulnerabilities.

Despite these advances, a critical gap remains in the literature. Comprehensive analyses that leverage the Global Health Security Index (GHSI) alongside advanced methodologies—such as entropy weighting, the Combined Compromise Solution (CoCoSo), and K-means clustering—are absent for non-EU European countries. Existing studies are limited by their narrow temporal or geographic scopes, often relying on single-year datasets or isolated regional frameworks. As a result, they lack longitudinal perspectives and omit comparative evaluations between non-EU European nations and other global regions. Furthermore, the synergistic potential of hybrid methodologies remains unexplored in this context.

This study addresses those significant research gaps by introducing a multidimensional framework that integrates Entropy, CoCoSo, and K-means clustering to assess the effectiveness of health security in 27 non-EU European countries between 2017 and 2021. By applying this innovative framework, this study provides a comprehensive examination of the health security landscape across these nations, offering invaluable insights into the state of their health systems during this period.

The primary objectives of this study are:

• To discern the most critical areas of focus and emerging patterns in health security outcomes within non-EU countries, thereby informing targeted interventions.
• To evaluate the HeS indicators of non-EU countries against those of the African and Eastern Mediterranean regions, enabling a nuanced understanding of their relative performance.
• To categorize and compare the health security performance of non-EU countries, facilitating a deeper analysis of their strengths and weaknesses.
• To identify distinct health security profiles among the countries studied, thereby enhancing our understanding of the factors contributing to performance variations and informing evidence-based policy decisions.

To achieve these objectives, the research undertakes a temporal analysis of health security trends using 2019, 2021, and aggregated 2017–2021 datasets, tracking evolving vulnerabilities and policy needs. The framework combines entropy weighting to assign indicator priorities, the CoCoSo for multi-criteria ranking, and K-means clustering to group countries into distinct clusters reflecting health security capacities. Comparative analysis with African and EMR systems further contextualizes disparities and shared challenges, informing tailored intervention strategies. Collectively, these objectives aim to deliver a nuanced understanding of health security dynamics and inform targeted, evidence-based interventions in non-EU European contexts.

## 2. Methods

This study employed a four-stage methodological framework to systematically assess health security performance. The following section delineates each stage in detail, alongside the cohesive integration of analytical techniques that unified the process.

### 2.1 Health security indicator compilation

This study utilizes 2019 and 2021 GHSI datasets to assess health security capacities across six domains: (1) Prevention, (2) Detection & Reporting, (3) Rapid Response, (4) Health System Resilience, (5) Compliance with International Norms, and (6) Risk Environment ^20^ (Global Health Security Index, Global Health Security Index Data Model and Report. (2021). Available at https://ghsindex.org/report-model/). The Global Health Security Index (GHSI) was selected for its established role in benchmarking global pandemic preparedness.

This study focuses on 27 non-EU European countries: Albania, Andorra, Armenia, Azerbaijan, Belarus, Bosnia and Herzegovina, Georgia, Iceland, Israel, Kazakhstan, Kyrgyz Republic, Liechtenstein, Moldova, Monaco, Montenegro, North Macedonia, Norway, Russia, San Marino, Serbia, Switzerland, Tajikistan, Turkey, Turkmenistan, Ukraine, the United Kingdom, and Uzbekistan. These nations, while geographically and culturally connected to Europe to varying degrees, operate outside the EU’s political-economic framework, presenting unique health security governance challenges and opportunities.

Nine performance metrics for six Global Health Security Index (GHSI) indicators (2019, 2021, and aggregated 2017–2021 data) were extracted for non-EU European countries, African, and Eastern Mediterranean (EMR) regions from the Global Health Security Index. ^20^ The non-EU metrics were evaluated against published analyses of Africa ^21^ and the EMR, ^22^ enabling cross-regional entropy-based comparisons of health security priorities, disparities, and temporal shifts.

For dynamic cross-national rankings and clustering, the non-EU data were analyzed using an integrated Entropy-CoCoSo (Combined Compromise Solution) and K-means model, categorizing countries into five risk clusters (high to dangerous). Trends, shared strengths, and weaknesses among clustered non-EU countries were assessed by analysing average scores across all 34 sub-indicators (2017–2021).

Comprehensive data are provided in Tables 1–11 (Sheet 1) of the supplementary software (MCDM-ML Based Tool for Health Security Optimization in Non-EU Countries Comprehensive Assessment and Visualization Workflow.xlsm.) as well as in the first section of the supplementary dataset file (The data and materials supporting the results and analyses of the paper.pdf ). ^38^ Source code and datasets are available at [ https://github.com/ProfAdelAbdulsalam/Supplementary-material--Software/tree/V2.0.1] and archived via [ https://doi.org/10.5281/zenodo.15185666]. ^38^

### 2.2 Assessing cross-regional variations in health security determinants: Entropy-driven estimation of criterion weights

The entropy method, rooted in information theory. ^39^ As a foundational approach in MCDM, it assigns objective weights to criteria, minimizing subjective bias in decision analysis. By measuring the uncertainty or “informational value” within a decision matrix, it quantifies data variability to assign higher weights to attributes with greater divergence, ensuring decisions reflect empirical patterns rather than subjective biases. ^40^ This data-driven approach eliminates reliance on expert opinions, making it particularly valuable in scenarios where impartiality is critical or expert input is unavailable. ^41^ Its versatility extends to handling diverse data types, including exact values and intervals, enhancing its applicability across fields like resource allocation, healthcare, and health security assessment. ^21^ While the entropy method’s effectiveness hinges on data quality, its adaptability and theoretical foundation solidify its role as a cornerstone in modern MCDM systems, bridging algorithmic precision with practical health security decision-making demands. ^21, 22^

The entropy method in this study follows a systematic five-step framework to derive objective criterion weights, progressing from data organization to weight computation. Below is a detailed breakdown ^21, 39^:

• Step 1. Decision Matrix Construction:

The process begins by structuring a decision matrix (G), as defined in Equation (1), where rows represent m countries and columns correspond to n health security indicators. Each element $g_{\mathrm{ij}}$ denotes the performance score of country i for indicator j:

$$G_{\mathrm{ij}}=\begin{array}{ccc} g_{11} & g_{12} & \ldots .g_{1n}\\ \ldots & .. & \ldots .\ldots ..\\ g_{m1} & g_{m2} & \ldots .g_{\mathrm{mn}} \end{array}\;$$ (1)

• Step 2. Normalization as follows:

$$n_{\mathrm{ij}}=\frac{g_{\mathrm{ij}}}{\sum_{i=1}^{m}g_{\mathrm{ij}}}\;$$ (2)

• Step 3. Entropy Calculation

For the *j*-th indicator, the entropy measure is calculated using Equation (3) to quantify its information content within the dataset, where lower entropy signifies higher informational utility.

$${\mathrm{Ent}}_{j}=-\left(\frac{1}{\ln\left(m\right)}\right)\sum_{i=1}^{m}n_{\mathrm{ij}}*\ln\;n_{\mathrm{ij}},,\forall j.$$ (3)

• Step 4. Divergence and Weight Derivation

The relative importance of each criterion is determined by its divergence $\left(1-{\mathrm{Ent}}_{j}\right)$ , which is normalized to compute final weights $W_{j}$ ( Equation 4). Indicators with greater variability receive higher weights:

$$W_{j}=\frac{1-{\mathrm{Ent}}_{j}}{\sum_{j=1}^{n}1-{\mathrm{Ent}}_{j}},\forall j.$$ (4)

The research expanded its examination of Health Security (HeS) indicators beyond non-European Union countries, with a particular focus on Africa and the Eastern Mediterranean Region (EMR). Employing the entropy method across nine iterations (encompassing 3 regions and 3 time periods: 2019, 2021, and 2017–2019), the study identified region-specific factors that impact health security. This methodological approach ensured consistency, facilitated cross-regional comparisons, and supported the development of targeted policy recommendations while maintaining global applicability.

Detailed calculations for this step are readily available in (Sheet 2.1 (a, b, c), (Sheet 2.2 (a, b, c), and (Sheet 2.3 (a, b, c)) of the supplementary software (MCDM-ML Based Tool for Health Security Optimization in Non-EU Countries Comprehensive Assessment and Visualization Workflow.xlsm.). ^38^ Source code and datasets are available at [ https://github.com/ProfAdelAbdulsalam/Supplementary-material--Software/tree/V2.0.1] and archived via [ https://doi.org/10.5281/zenodo.15185666]. ^38^

### 2.3. Prioritizing Non-EU countries using an integrated entropy-CoCoSo method

This study employs the Combined Compromise Solution (CoCoSo) method, a robust multi-criteria decision-making (MCDM) technique, to evaluate and rank non-EU countries based on a set of predefined criteria. The CoCoSo method’s strength lies in its ability to integrate three aggregation strategies – weighted sum model (WSM), weighted product model (WPM), and an exponential compromise strategy – to derive a balanced ranking of alternatives. ^42^ By synthesizing outcomes from distinct mathematical models, this approach ensures robustness and reduces the bias inherent in single-aggregation techniques. ^41^

In addition to its effective application in evaluating health security practices, as highlighted in Study, ^23^ numerous studies have demonstrated the efficiency of integrating methods like entropy in various domains. For instance, Research ^33^ evaluated the progress of Indian Union Territories (UTs) toward achieving Sustainable Development Goals (SDGs) using the SDG India Index 3.0 (2020–2022 dataset). To tackle the contextual variability inherent in sustainability frameworks, the authors employed a hybrid multicriteria decision-making (MCDM) model that combined Shannon Entropy—leveraging data variability to objectively weight SDG indicators—and the COCOSO (Combined Compromise Solution) method, which ranked UTs by balancing multiple compromise scores.

The integrated Shannon Entropy-COCOSO framework effectively quantified disparities in SDG implementation across the Indian Union Territories. The entropy weighting mechanism allowed for the objective prioritization of indicators based on their variability, while the COCOSO method facilitated the ranking of regions through comprehensive multi-criteria compromise scores. This analysis revealed notable extremes, with Chandigarh ranked highest and Dadra & Nagar Haveli/Daman & Diu positioned lowest in terms of SDG achievement. The model’s robust validation highlights its utility in identifying context-specific gaps, thereby guiding targeted interventions aimed at aligning underperforming regions with Agenda 2030.

Similarly, Study ^43^ developed collagen-based packaging films (CSP and CSC) from leather and food waste and evaluated their performance through a hybrid Entropy-CoCoSo framework. This framework utilized entropy weighting to objectively prioritize material properties, such as water solubility and biodegradability, while CoCoSO aggregated multi-criteria scores to rank disparities between different film formulations.

These examples underscore the powerful advantages of integrating methodologies, as they not only enhance analytical robustness but also provide nuanced insights that are essential for formulating effective, targeted strategies in diverse fields.

In this study, we implemented the CoCoSo algorithm in four stages, assigning criteria weights using the entropy method to ensure objectivity, as validated in recent MCDM frameworks. ^33, 43^ The CoCoSo method was chosen for its unique ability to handle trade-offs between conflicting criteria while providing stable and interpretable rankings, as demonstrated by its superior performance in comparative analyses compared to other MCDM methods. ^35, 41^ The key steps of the CoCoSo method are outlined as follows ^42^:

• Step 1: Constructing the Decision Matrix (G) and Normalization

The method begins with the decision matrix G = [ $g_{\mathrm{ij}}$ ], where $g_{\mathrm{ij}}$ represents the performance score of alternative i for criterion j, as shown in Equation (1). Normalization is applied to eliminate scale differences using Equation (5):

$$n_{\mathrm{ij}}=\left\{ \begin{array}{c} \;\frac{g_{\mathrm{ij}}-g_{j}^{-}}{g_{j}^{+}-g_{j}^{-}},\text{if}\;C_{j}\;\text{is}\;a\;\text{benefit criterion}\\ \frac{g_{\mathrm{ij}}-g_{j}^{+}}{g_{j}^{-}-g_{j}^{+}},\text{if}\;C_{j}\;\text{is}\;a\;\text{cost criterion} \end{array}\right.,$$ (5)

Here, $g_{j}^{+}$ and $g_{j}^{-}$ denote the maximum and minimum values of criterion j, respectively, across all countries i.

• Step 2. Computing the weighted comparability sequences as follows:

Additive Weighted Sequence ( Equation 6):

$${\mathrm{CS}}_{i}=\sum_{j=1}^{n}w_{j}n_{\mathrm{ij}},$$ (6)

Exponential Weighted Sequence ( Equation 7):

$$P_{i}=\sum_{j=1}^{n}{\left(n_{\mathrm{ij}}\right)}^{w_{j}},$$ (7)

Here, $w_{j}$ is the weight of criterion j, derived using the entropy method to ensure objectivity.

• Step 3: Calculating the relative importance scores.

Three appraisal scores are calculated to evaluate alternatives: Normalized Arithmetic Mean ( Equation 8); Relative Superiority Score ( Equation 9); and Balanced Compromise Score ( Equation 10).

$$k_{\mathrm{ia}}=\left(P_{i}+S_{i}\right)/\sum_{i=1}^{m}\left(P_{i}+S_{i}\right),$$ (8)

$$k_{\mathrm{ib}}=\left(S_{i}/\min\;S_{i}\right)+\left(P_{i}/\min\;P_{i}\right),$$ (9)

$$k_{\mathrm{ic}}=\frac{\left(\lambda \left(S_{i}\right)+\left(1-\lambda \right)P_{i}\right)}{\left(\lambda \;\left(\max\;S_{i}\right)+\left(1-\lambda \right)\max\;P_{i}\right)},$$ (10)

The parameter λ (0 ≤ λ ≤ 1) allows decision-makers to adjust the influence of S _i_ (additive) and P _i_ (exponential). A default λ = 0.5 ensures equal weighting.

• Step 4: Composite Score and Ranking

The final composite score C _i_ ( Equation 11) combines all three appraisal scores:

$$C_{i}=\left({\left(k_{\mathrm{ia}}+k_{\mathrm{ib}}+k_{\mathrm{ic}}\right)}^{\frac{1}{3}}\right)+\left(\frac{\left(k_{\mathrm{ia}}+k_{\mathrm{ib}}+k_{\mathrm{ic}}\right)}{3}\right),$$ (11)

Alternatives are ranked in descending order of $C_{i}$ , with higher values indicating superior performance.

This study applied the entropy-CoCoSo algorithm to rank Non-EU countries across three time periods (2019, 2021, 2017–2019), evaluating preparedness and capabilities for addressing global health challenges.

Detailed calculations for this step are readily available in (Sheet 3 (a, b, c)) of the supplementary software (MCDM-ML Based Tool for Health Security Optimization in Non-EU Countries Comprehensive Assessment and Visualization Workflow.xlsm.). ^38^ Source code and datasets are available at [ https://github.com/ProfAdelAbdulsalam/Supplementary-material--Software/tree/V2.0.1] and archived via [ https://doi.org/10.5281/zenodo.15185666]. ^38^

### 2.4 Clustering

K-means clustering is a centroid-based machine learning method that categorizes unlabelled data into a specified number of clusters by iteratively optimizing the proximity of data points to cluster centroids. ^36^ By grouping observations into homogeneous clusters, this algorithm maximizes similarity within each group while minimizing variance between them.

The process begins by initializing K cluster centroids, with each data point assigned to the nearest centroid using distance metrics such as Euclidean distance, forming provisional clusters. Subsequently, the centroids are recalculated as the mean of all points within their respective clusters, and this iterative cycle of assignment and centroid updates continues until the centroids stabilize (with minimal positional change) or a maximum iteration threshold is reached. ^36^

Notably, building upon the foundational work highlighted in previous studies and its successful application in prior health security analyses to classify countries in various regions, ^21– 23^ K-means clustering’s simplicity and computational efficiency make it well-suited for this analysis. ^21^ By categorizing countries into homogeneous clusters based on key health security indicators, we can effectively discern patterns and disparities that may not be immediately evident through other analytical methods. ^22^ Specifically, we utilized K-means clustering to partition the data into stratified groups, allowing for a nuanced examination of varying health security profiles. ^23^ This approach enables us to visualize and contextualize the differences between countries, facilitating a targeted analysis of regional vulnerabilities. ^21– 23^

Furthermore, by employing clustering, we can track shifts in health security performance over time, providing valuable insights for policymakers to implement tailored interventions. ^21– 23^ In combining this method with advanced multi-criteria decision-making techniques—such as entropy weighting and the CoCoSo method—we reinforce the robustness of our findings. The integration of clustering exemplifies a methodological synergy that not only enhances our analytical rigor but also empowers stakeholders with actionable insights derived from complex data landscapes. ^21– 23^

In our study, we adopted a five-level categorization framework (1–5) to evaluate regional performance across countries, with a rating scale spanning from 1 (high) to 5 (dangerous), and intermediate tiers labelled as 2 (safe), 3 (moderate), and 4 (alert), allowing for a granular assessment of regional disparities. To classify non-EU nations, we repeated the clustering procedure across three independent iterations, each corresponding to outcomes derived from a composite ranking methodology applied to three distinct datasets.

To facilitate the clustering process of Non-EU countries across three time periods (2019, 2021, 2017–2019), we utilized an open source software, ^44^ a freely available tool for segmentation tasks. ( https://www.clusteranalysis4marketing.com/a-marketers-guide-to-cluster-analysis/free-download/). Then the clustering results were integrated into our developed software for further analysing process, detailed calculations for this step are readily available in (Sheet 4 (a, b, c)) of the supplementary software (MCDM-ML Based Tool for Health Security Optimization in Non-EU Countries Comprehensive Assessment and Visualization Workflow.xlsm.). ^38^ Source code and datasets are available at [ https://github.com/ProfAdelAbdulsalam/Supplementary-material--Software/tree/V2.0.1] and archived via [ https://doi.org/10.5281/zenodo.15185666]. ^38^

### 2.5 Software

For this study, a simple Excel-based software tool was developed to systematically assess Health Security (HeS) inequalities across non-EU European countries. ^38^

Source code and datasets are available at [ https://github.com/ProfAdelAbdulsalam/Supplementary-material--Software/tree/V2.0.1] and archived via [ https://doi.org/10.5281/zenodo.15185666]. ^38^

The Main Menu of the software tool serves as the central navigation interface, guiding users through the sequential workflow of the integrated Multi-Criteria Decision-Making (MCDM) - Machine Learning methodology. It illustrates the Four-step implementation and analytical process described in this research.

For Visualization, this tool provides access to tabular and graphical outputs, including entropy weights, rankings, and cluster patterns. In addition, the tool’s structured layout mirrors the research methodology, enabling seamless navigation between data input, computational analysis, and result interpretation. Interactive hyperlinks to corresponding sheets or modules ensure a user-friendly experience while maintaining alignment with the paper’s technical framework.

## 3. Results

### 3.1 Entropy-driven assessment of HeS priorities

**Figure 1.**
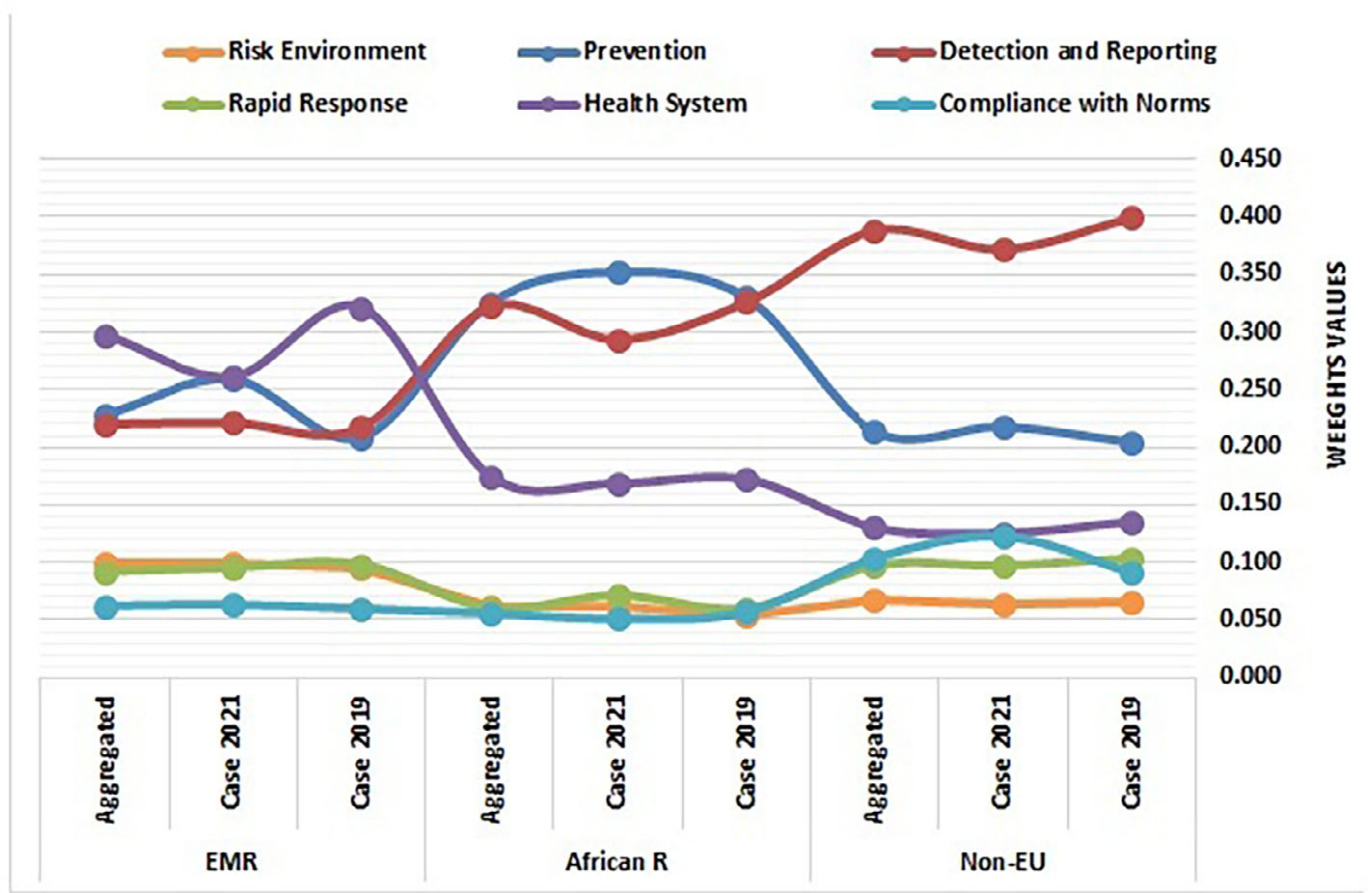
Entropy-weighted prioritization of health security dimensions across non-EU countries, EMR, and African Region, 2017–2021 (Source: Authors). This figure illustrates the entropy-weighted prioritization of health security dimensions across non-EU European countries, the Eastern Mediterranean Region (EMR), and the African Region (2017–2021). The figure compares the relative weights of six Global Health Security Index (GHSI) indicators—Prevention, Detection & Reporting, Rapid Response, Health System, Compliance with Norms, and Risk Environment—across regions and timeframes.

Table 1 presents the entropy-weighted prioritization of health security dimensions across regions. Weights for six GHSI indicators in non-EU Europe, Africa, and the EMR (2019, 2021, aggregated 2017–2021), It compares the relative weights of six Global Health Security Index (GHSI) indicators. Together with Figure 1, this table offers critical insights into regional prioritization patterns of HeS indicators. Figure 1 illustrates the comparative distribution of weights across HeS dimensions for non-EU countries, the African Region, and the EMR over three timeframes: 2019, 2021, and the aggregated period of 2017–2021. Complementing this analysis, Table 2 illustrates the comparative entropy weight changes (2019–2021) across regions. Positive/negative values indicate increased/decreased prioritization of indicators over time.

**Table 1.** Entropy-weighted prioritization of health security dimensions across non-EU countries, Eastern Mediterranean Region (EMR), and African Region.

Year	Region	Weights values					
		Prevention	Detection and reporting	Rapid response	Health system	Compliance with norms	Risk environment
**2019**	Non-EU European countries	0.204	0.399	0.104	0.135	0.091	0.066
**2021**		0.218	0.372	0.098	0.126	0.123	0.064
**Aggregated**		0.214	0.388	0.097	0.130	0.104	0.067
**2019**	African Region	0.330	0.326	0.060	0.172	0.057	0.055
**2021**		0.352	0.294	0.072	0.169	0.051	0.063
**Aggregated**		0.325	0.322	0.061	0.175	0.056	0.061
**2019**	Eastern Mediterranean regions	0.209	0.217	0.098	0.322	0.060	0.095
**2021**		0.259	0.221	0.096	0.261	0.063	0.099
**Aggregated**		0.228	0.220	0.092	0.298	0.063	0.099

**Table 2.** Comparative entropy weight changes (2019–2021) across non-EU, African, and Eastern Mediterranean regions.

Region	Weights values					
	Prevention	Detection and reporting	Rapid response	Health system	Compliance with norms	Risk environment
**Non-EU European countries, **	0.014	-0.027	-0.006	-0.010	0.031	-0.001
**African Region**	0.023	-0.032	0.011	-0.004	-0.006	0.008
**Eastern Mediterranean regions**	0.051	0.004	-0.003	-0.060	0.004	0.004

### 3.2 Health security rankings and cluster-based interventions

**Figure 2.**
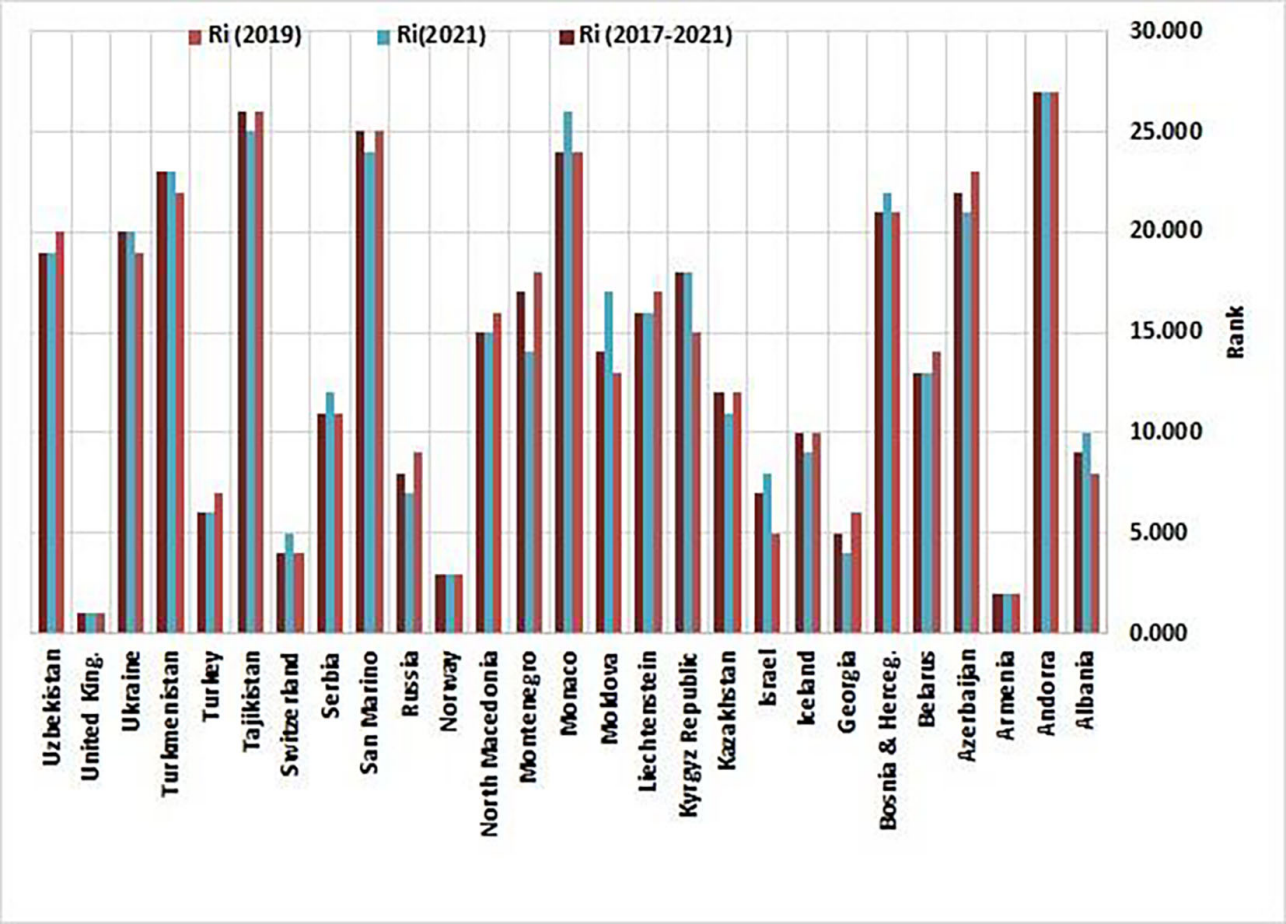
Health Security Preparedness Rankings of Non-EU countries (Source: Authors). This bar chart illustrates the Health Security Preparedness Rankings of non-EU European countries, encompassing the years 2019, 2021, and the entire period of 2017-2021. Countries are ranked based on their Composite CoCoSo scores, with higher values indicating superior performance. It also reflects temporal shifts in health security capabilities.

**Figure 3.**
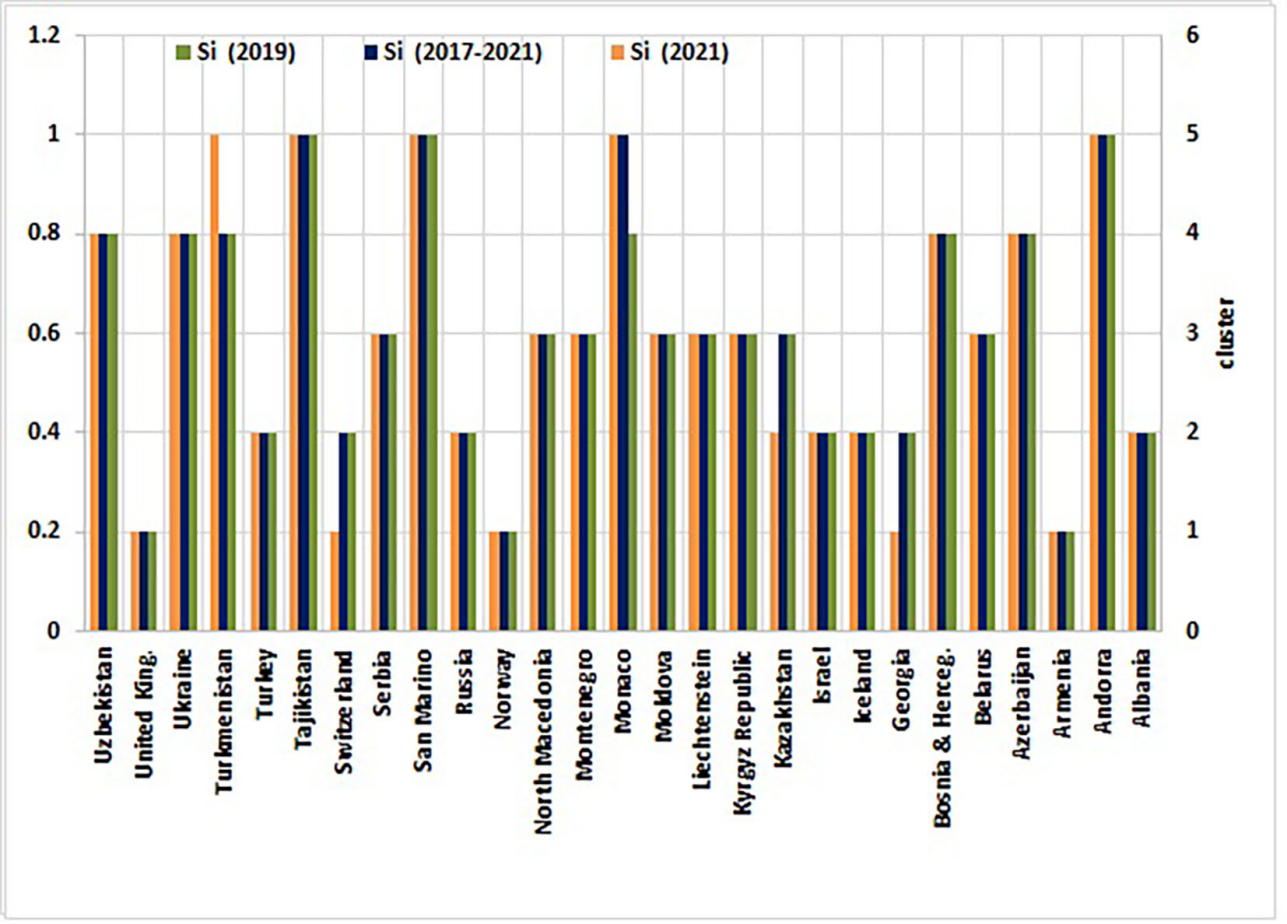
Health security performance clustering results of Non-EU countries (Source: Authors). This figure illustrates the K-Means Clustering results of non-EU European countries, encompassing the years 2019, 2021, and the entire period of 2017-2021. Countries are grouped into five clusters based on their health security profiles, with Cluster 1 representing exemplary performance and Cluster 5 representing critical performance. The figure also reveals patterns of similarity and dissimilarity among countries in terms of their health security capabilities.

Table 3 outlines the performance metrics and comparative standings of non-EU countries derived from the Entropy-CoCoSo HeS methodology. For three intervals—2019, 2021, and the cumulative 2017–2021 timeframe—the table displays computed scores (Ci) and their ordinal positions (Ri). Each country is further classified into one of five performance tiers (Si), where S1denotes optimal outcomes (‘High Performance’) and S5signals critical deficiencies (‘Dangerous Level’). The data also track fluctuations in rankings and tier placements between 2019 and 2021, illustrating shifts in national progress or regression. Figure 2 visually maps the ranked outcomes, while Figure 3 depicts the spatial distribution of clusters across this group of countries.

**Table 3.** Entropy-CoCoSo HeS scores, rankings, and cluster classifications.

	Country	2019			2021			The whole period (2017-2021)		
		Ki	Ri	Si	Ki	Ri	Si	Ki	Ri	Si
***1***	Albania	3.088	8	2	2.231	10	2	2.464	9	2
***2***	Andorra	1.162	27	5	1.216	27	5	1.148	27	5
***3***	Armenia	4.388	2	1	3.066	2	1	3.439	2	1
***4***	Azerbaijan	2.039	23	4	1.681	21	4	1.747	22	4
***5***	Belarus	2.572	14	3	2.135	13	3	2.214	13	3
***6***	Bosnia & Herzegovina	2.093	21	4	1.652	22	4	1.754	21	4
***7***	Georgia	3.247	6	2	2.692	4	1	2.803	5	2
***8***	Iceland	2.893	10	2	2.265	9	2	2.417	10	2
***9***	Israel	3.285	5	2	2.327	8	2	2.598	7	2
***10***	Kazakhstan	2.680	12	3	2.192	11	2	2.291	12	3
***11***	Kyrgyz Republic	2.570	15	3	1.993	18	3	2.130	18	3
***12***	Liechtenstein	2.518	17	3	2.037	16	3	2.136	16	3
***13***	Moldova	2.673	13	3	2.032	17	3	2.190	14	3
***14***	Monaco	1.712	24	4	1.350	26	5	1.436	24	5
***15***	Montenegro	2.372	18	3	2.116	14	3	2.134	17	3
***16***	North Macedonia	2.540	16	3	2.088	15	3	2.176	15	3
***17***	Norway	3.890	3	1	2.751	3	1	3.066	3	1
***18***	Russia	3.030	9	2	2.404	7	2	2.545	8	2
***19***	San Marino	1.413	25	5	1.373	24	5	1.397	25	5
***20***	Serbia	2.794	11	3	2.137	12	3	2.297	11	3
***21***	Switzerland	3.665	4	2	2.678	5	1	2.947	4	2
***22***	Tajikistan	1.386	26	5	1.363	25	5	1.373	26	5
***23***	Turkey	3.151	7	2	2.424	6	2	2.601	6	2
***24***	Turkmenistan	2.081	22	4	1.441	23	5	1.550	23	4
***25***	Ukraine	2.243	19	4	1.753	20	4	1.804	20	4
***26***	United Kingdom	4.467	1	1	3.208	1	1	3.557	1	1
***27***	Uzbekistan	2.217	20	4	1.835	19	4	1.902	19	4

## 4. Discussion

### 4.1 Comparative weighting of health security indicators: Non-EU, Africa, and EMR perspectives

**4.1.1. Discussion of Non-EU Countries’ health security priorities**

The health security landscape among non-European countries reveals significant trends and variations in prioritization, as evident in Table 1 and Figure 1. A notable finding is the consistent emergence of detection & reporting as the most influential HeS indicator, with weights ranging from 0.372 to 0.399 across different time periods. This underscores the critical importance of surveillance capabilities and data transparency, which are essential components in identifying and managing public health threats. The slight decline in its weight by 2021, from 0.399 to 0.372, may reflect challenges in `maintaining robust reporting mechanisms amidst the complexities posed by emerging health crises, such as the COVID-19 pandemic, which exposed weaknesses in global surveillance systems. ^45^ This decline highlights the need for sustained efforts to strengthen surveillance capabilities.

The increasing weight of prevention, from 0.204 to 0.218 over time, signifies a growing recognition of the need for pre-emptive measures, such as vaccination and overall public health preparedness. This shift resonates with global trends that emphasize the necessity of robust preventive strategies to mitigate the impact of future health emergencies, as emphasized by WHO initiatives aimed at enhancing vaccine coverage globally. ^46^ However, the disparities observed in vaccination rates and public health preparedness among non-EU countries hint at persistent inequities that require concerted efforts to address.

In contrast, compliance with international norms shows a significant increase from 0.091 to 0.123 post-2019, indicating emerging regulatory gaps or uneven adherence to established health standards. This trend may reflect the ongoing developments in international health regulations, such as the International Health Regulations (IHR), and recent reforms in Europe, which have urged nations to align their practices with global and regional standards. ^47^ The rising focus on compliance underscores the necessity for stricter regulatory enforcement to curb health security vulnerabilities that could compromise global health outcomes.

Furthermore, indicators such as Rapid Response (0.097–0.104) and Health System (0.126–0.135) are less influential, suggesting systemic weaknesses in emergency coordination and healthcare infrastructure across these countries. The relative stagnation of these areas points to the challenges faced by non-EU states in developing swift and effective responses to health emergencies, a concern that aligns with findings from global health security initiatives highlighting the importance of strengthening health systems in those countries. ^48^

Additionally, the Risk Environment indicator remains minimally influential (~0.06–0.067), reflecting persistent vulnerabilities faced by non-EU countries concerning external threats, such as climate change or geopolitical instabilities. This finding parallels discussions in public health literature concerning the interplay between environmental factors and health security, suggesting that a multi-faceted approach that encompasses both health and environmental policies is critical. ^49^

The aggregated weight stability across these indicators signifies a sustained focus on surveillance and prevention, while the rising weights of compliance underscore the urgent need for enhanced regulatory measures. To address these challenges, policymakers must prioritize the harmonization of detection systems, the scaling up of preventive measures, and addressing compliance gaps to reduce disparities among countries. Moreover, fostering collaboration and knowledge sharing among nations can bolster resilience against emergent health threats, significantly aligning with international efforts to build a more robust global health security framework. ^50^ Through such concerted actions, non-EU countries can better navigate the complexities of health security and enhance their overall preparedness for future public health challenges.

**4.1.2 Comparative analysis of Non-EU and African health security priorities**

Non-EU countries and the African region exhibit stark contrasts in health security prioritization. In non-EU states, detection & reporting dominates (0.372–0.399), reflecting significant disparities in surveillance systems and data-sharing capabilities, while prevention (0.204–0.218) plays a secondary role. Conversely, Africa prioritizes prevention (0.325–0.352) as its top indicator, highlighting variability in pre-emptive measures like vaccine access and sanitation infrastructure, with detection & reporting declining post-2019 (0.326 → 0.294), ^21^ signalling converging surveillance challenges. Non-EU’s compliance with norms surged (0.091 → 0.123), pointing to emerging regulatory gaps, whereas Africa’s compliance remains stagnant (~0.05–0.06), suggesting systemic neglect of health standards. Both regions show weak differentiation in Rapid Response (non-EU: 0.097–0.104; Africa: 0.060–0.072), exposing shared deficiencies in emergency coordination. ^51^ However, Africa’s health system weights (0.169–0.175) surpass non-EU’s (0.126–0.135), indicating greater disparities in healthcare infrastructure across African nations. ^52^ Risk Environment is uniformly low (~0.06), underscoring neglected climate or geopolitical vulnerabilities in both regions. These contrasts necessitate tailored policies: non-EU should harmonize surveillance networks and enforce compliance, while Africa must scale preventive infrastructure and healthcare investments. Cross-regional collaboration—leveraging non-EU’s surveillance expertise to bolster Africa’s detection gaps and Africa’s preventive models to inform non-EU strategies—could address shared weaknesses in crisis response and risk resilience.

**4.1.3 Comparative analysis of Non-EU and EMR health security priorities**

Non-EU countries and the Eastern Mediterranean Region (EMR) demonstrate divergent health security priorities. Non-EU nations prioritize detection & reporting (0.372–0.399) as the dominant indicator, reflecting variability in surveillance systems, while the EMR focuses overwhelmingly on health systems (0.261–0.322), highlighting disparities in healthcare infrastructure and workforce capacity. ^21^ Notably, the EMR’s prevention weight rises sharply (0.209 → 0.259) by 2021, nearing the non-EU’s emphasis (0.204–0.218), suggesting a growing recognition of pre-emptive measures like vaccine equity. However, Detection & Reporting remains far less influential in the EMR (0.217–0.221 vs. non-EU’s 0.372–0.399), indicating weaker differentiation in surveillance capabilities. Both regions share low weights for Rapid Response (non-EU: 0.097–0.104; EMR: 0.092–0.098), underscoring systemic gaps in emergency coordination. Compliance with norms is marginally higher in non-EU (0.091 → 0.123) compared to the EMR’s stagnant values (~0.06–0.063), suggesting non-EU faces emerging regulatory challenges, while the EMR neglects this domain. The EMR also shows slightly higher risk environment weights (~0.095–0.099 vs. non-EU’s ~0.06–0.067), signalling greater variability in political, and socioeconomically vulnerabilities.

**4.1.4 Non-EU health security dynamics: Cross-regional contrasts and emerging challenges in global health governance**

The examination of non-EU countries’ health security dynamics between 2019 and 2021 reveals significant contrasts and emerging challenges that shape global health governance. Notably, the increased prioritization of prevention (+0.014) and compliance with norms (+0.031) indicates a growing recognition among non-EU nations of the importance of pre-emptive measures and the need to address regulatory gaps. This trend aligns with a global movement towards enhancing preventive health strategies and compliance with international health regulations as essential components of health security. ^47^ The rising focus on compliance, in particular, underscores the importance of strengthening regulatory frameworks to ensure adherence to global health standards.

In contrast, the sharp decline in detection and reporting (-0.027) within non-EU countries echoes a similar trend observed in Africa (-0.032). This decline raises concerns about diminishing surveillance capabilities and transparency in reporting health threats. Notably, the Eastern Mediterranean Region (EMR) experienced a slight gain in detection (+0.004), highlighting a divergence in surveillance dynamics that underscores the necessity for tailored approaches in each region. The disparities in detection efforts reflect varying capacities for health monitoring and reporting, necessitating an urgent focus on enhancing the data infrastructure and capabilities across non-EU countries. ^53^

Furthermore, the loss of relevance in health system indicators (-0.010) among non-EU countries is particularly concerning. This trend diverges from Africa’s marginal decline (-0.004) and the EMR’s steep drop (-0.060), indicating a complex landscape of healthcare vulnerabilities. The systemic weaknesses faced by both non-EU and African nations pose significant challenges to their health security, revealing a pressing need for investment in healthcare infrastructure and systems. Conversely, the worsening infrastructure circumstances in the EMR post-pandemic highlight the urgent need for stabilization efforts, further emphasizing the regional disparities in health system resilience. ^22^

Interestingly, the rising focus on regulatory compliance in non-EU countries (+0.031) stands in contrast to Africa’s stagnant compliance (-0.006). This finding suggests a fragmented adherence to global health norms, signifying the need for improved mechanisms to ensure that all regions align with international standards. Such contrasts in compliance outcomes align with the assertions of, ^22^ advocating for a more cohesive and collaborative global approach to health governance, capable of bridging gaps and enhancing compliance across diverse contexts.

Despite these emerging improvements in compliance, all regions, including non-EU countries, continue to neglect rapid response capabilities, with non-EU exhibiting a decline of (-0.006). While Africa has shown a slight improvement in this domain (+0.011), the EMR has experienced a minimal drop (-0.003). Africa’s improvement hints at the potential development of emerging crisis coordination efforts, which could be furthered by leveraging gains in prevention and compliance. These insights indicate that while progress is being made, a holistic approach to emergency preparedness remains imperative across all regions.

Ultimately, these contrasting trends highlight the necessity for tailored strategies to address distinct regional challenges. Non-EU countries must find ways to balance the losses in surveillance capabilities with the gains in compliance, while African nations are encouraged to build upon their momentum in prevention. In the EMR, there is an urgent need to focus on stabilizing healthcare systems to prepare for future crises. By recognizing these disparities and aligning regional strategies with global health priorities, stakeholders can work towards reinforcing the resilience and effectiveness of health security frameworks worldwide. ^22, 23^

### 4.2 Interpreting regional disparities in health security rankings

The analysis of Health Security Index rankings among non-EU countries from 2019 to 2021 reveals a complex landscape of health security performance, with significant variations indicative of different responses to health security challenges and regional dynamics. As illustrated in Figure 2, thirteen countries (48.14%), including Belarus, Iceland, Kazakhstan, Liechtenstein, North Macedonia, San Marino, Tajikistan, Turkey, Uzbekistan, Azerbaijan, Georgia, Russia, and Montenegro, demonstrated improved rank values, signalling advancements in health security measures. These improvements underscore the effectiveness of targeted interventions and policy changes implemented in those nations, such as Montenegro’s successful health governance strategies and readiness to address public health threats. ^54^

In contrast, four countries (14.8%), including Andorra, Armenia, Norway, and the United Kingdom, maintained stable performance, indicating a consistent approach to health security that effectively mitigated the need for drastic policy shifts. However, a concerning 37% of countries, including Moldova, Israel, and the Kyrgyz Republic, saw declines in their rankings, which signals urgent areas for improvement in their health security frameworks. The declines in these countries are attributed to various factors, including the absence of comprehensive policies, inadequate biosecurity regulations, and insufficient accountability for international commitments.

Moldova’s decline, for instance, was characterized by a drop in rank due to the absence of policies concerning export/import restrictions and travel bans during infectious disease outbreaks, illustrating critical vulnerabilities in its health security preparedness. ^55^ Similarly, Israel faced significant reductions in foundational measures such as biosecurity regulations and immunization rates, leading to a decline in ranking. These gaps necessitate a re-evaluation of Israel’s health security infrastructure to enhance resilience and ensure effective responses to health crises.

Kyrgyzstan’s situation mirrors that of Moldova and Israel, as evidenced by similar declines in preparedness due to the absence of policies on travel restrictions and insufficient accountability for international commitments. ^56^ The 25-point decline in social stability further exacerbates the challenges faced by Kyrgyzstan in maintaining an effective health security framework. There is a critical need for Kyrgyzstan to prioritize health security improvements to better prepare for health emergencies and safeguard public health.

Collectively, these findings emphasize the need for a comprehensive understanding of the underlying causes contributing to the observed declines in health security rankings across non-EU countries. Targeted analyses are essential to develop effective strategies that can address these vulnerabilities, including enhancing regulatory frameworks, improving public health infrastructure, and fostering community engagement in health governance. ^57^ By acknowledging and addressing these critical areas, non-EU countries can bolster their health security frameworks, ultimately leading to stronger health outcomes and improved resilience against future public health threats.

### 4.3 Health security performance: Insights and patterns across five clusters

Using the final Entropy-CoCoSo assessment scores, non-EU countries were categorized into five distinct health security performance clusters during both individual sub-assessment years and the entire study period ( Table 4, Figure 3).

**Table 4.** Health security performance clusters of the non-EU-27 countries.

Cluster (Level)	2019	2021	The whole period (2017-2021)
** 1 (High)**	Armenia, Norway, United Kingdom	Armenia, Norway, United Kingdom, Georgia, Switzerland	Armenia, Norway, United Kingdom
**2 (Safe)**	Georgia, Switzerland, Albania, Iceland, Israel, Russia, Turkey, Kazakhstan	Albania, Iceland, Israel, Russia, Turkey, Kazakhstan	Albania, Georgia, Iceland, Israel, Russia, Switzerland, Turkey
**3 (Intermediate)**	Belarus, Kyrgyz Republic, Liechtenstein, Moldova, Montenegro, North Macedonia, Serbia	Belarus, Kyrgyz Republic, Liechtenstein, Moldova, Montenegro, North Macedonia, Serbia	Belarus, Kazakhstan, Kyrgyz Republic, Liechtenstein, Moldova, Montenegro, North Macedonia, Serbia
**4 (Warning)**	Azerbaijan, Bosnia & Herzegovina, Ukraine, Uzbekistan, Turkmenistan, Monaco	Azerbaijan, Bosnia & Herzegovina, Ukraine, Uzbekistan	Azerbaijan, Bosnia & Herzegovina, Turkmenistan, Ukraine, Uzbekistan
**5 (Dangerous)**	Andorra, San Marino, Tajikistan	Turkmenistan, Andorra, Monaco, San Marino, Tajikistan	Andorra, Monaco, San Marino, Tajikistan

Table 4 and Figure 3 categorize countries into five clusters (High, Safe, Intermediate, Warning, and Dangerous) using K-means clustering of composite Entropy-CoCoSo scores. Cluster assignments reflect temporal stability or shifts in health security capabilities across the study period. These clusters, spanning from High to Dangerous, delineate varying levels of health security preparedness, offering a stratified perspective on systemic capabilities across non-EU countries.

***4.3.1 Bridging gaps in Non-EU health security: Strategic interventions for cluster-specific risks***

**Figure 4.**
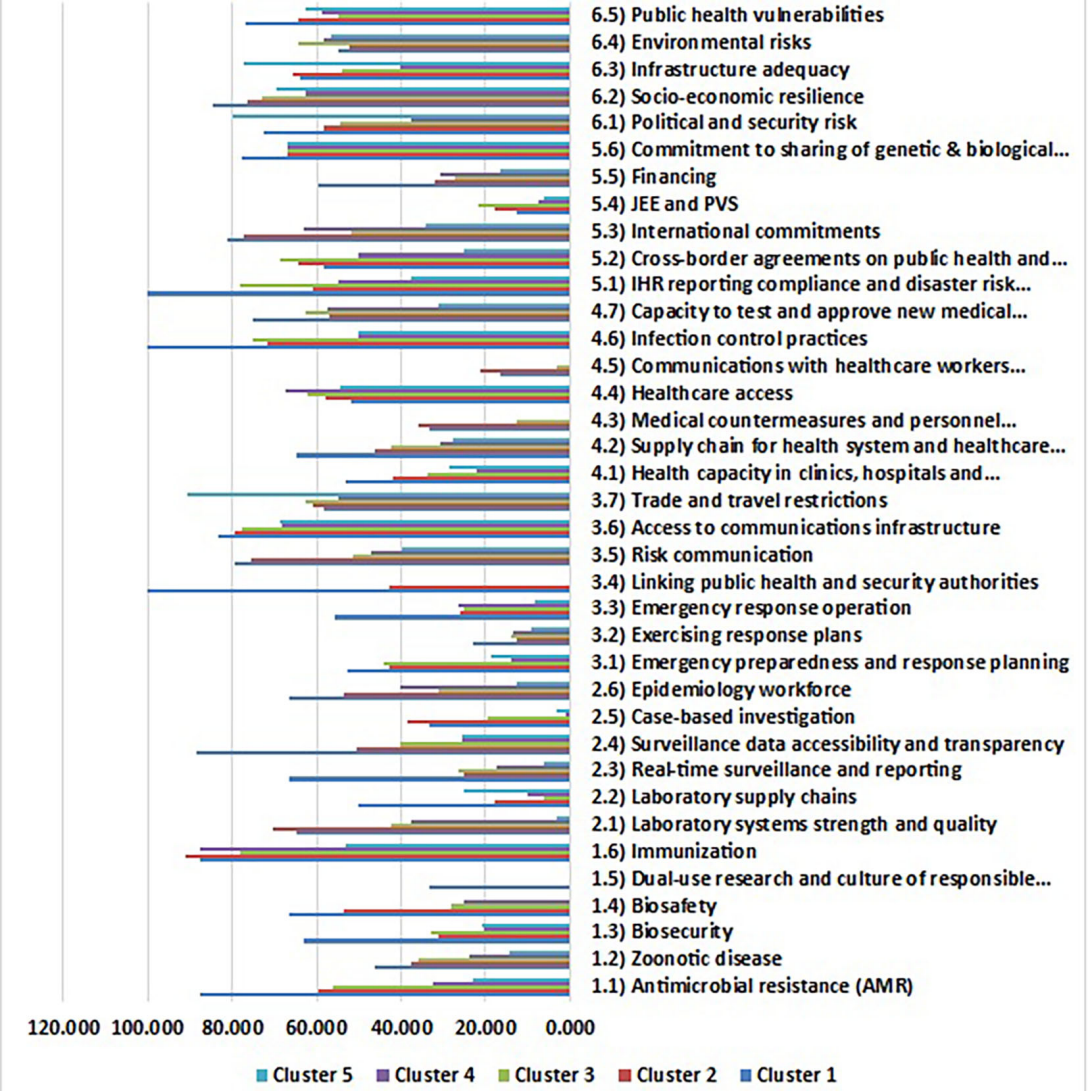
Average health security performance scores across the five clusters of non-EU countries (Source: Authors). This figure compares the mean scores of six HeS sub-indicators for each cluster (High, Safe, Intermediate, Warning, and Dangerous). Scores range from 0 to 100, with higher values indicating stronger performance. The visualization highlights disparities in HeS capabilities across clusters. Source: Authors’ analysis based on GHSI 2021 data.

The analysis of health security indicators across five distinct clusters of non-EU countries reveals marked disparities and critical interdependencies that necessitate strategic interventions tailored to specific risks. Within the realm of prevention indicators, as shown in Figures 3, and 4, the high-performance cluster (Cluster 1), represented by countries like Armenia, Norway, and the United Kingdom, averaged a score of 87.48 in antimicrobial resistance (AMR) preparedness. This stands in stark contrast to Cluster 5 (Dangerous-Level), which includes nations such as Andorra, Monaco, San Marino, and Tajikistan, scoring significantly lower at 62.50. These disparities underscore the pressing need for targeted capacity-building initiatives in lower-performing regions. ^23^

Furthermore, the universal suboptimal readiness for zoonotic diseases across all clusters, particularly declining to a critical low of 14.53 in Cluster 5, highlights a collective vulnerability to emerging infectious diseases. The strikingly low biosafety and biosecurity metrics—21 and 0, respectively, in Cluster 5, as compared to scores of 63 and 67 in Cluster 1—point to systemic gaps that require urgent attention. The absence of dual-use research and responsible science practices outside of Cluster 1 further emphasizes the necessity for enhanced regulations and education throughout the entire region.

The evaluation of detection and reporting capabilities reveals a concerning downward trajectory of surveillance accessibility, laboratory systems, and epidemiological workforce capacity across clusters. The significant decline from an average score of 88.33 to 25 in surveillance accessibility reflects deteriorating capacities to monitor and respond to health threats effectively. Laboratory supply chains remain critically low, with Cluster 1 being the only group to achieve a score above 25. The extreme variance in epidemiological workforce metrics observed in Cluster 2 (1,148) highlights intra-cluster heterogeneity that requires more granular investigations to address the differences in capacity and deployment.

Rapid response capabilities exhibit stark disparities, with Cluster 1 leading at 52.80 while Clusters 4 and 5 lag behind with scores of 14.17 and 18.75, respectively. The universally inadequate testing of response plans indicates a significant gap in preparedness across all clusters. Notably, the integration of public health and security authorities is present only in Cluster 1 (100), emphasizing the crucial role of inter-agency collaboration in emergency preparedness.

The analysis of health system capacity reveals substantial gradients in health capacity and supply chain resilience, with scores ranging dramatically from 53.08 in some clusters down to 22.12 in others. The critical deficiencies in medical countermeasures—registered only at nominal levels in Clusters 1 and 2—point towards the need for focused investments in essential health resources and response mechanisms. Additionally, while infection control measures excel in Cluster 1, the moderate implementation seen in Clusters 4 and 5 indicates areas needing improvement.

Compliance metrics across clusters showed systemic shortcomings, particularly concerning joint external evaluations (JEE) and performance of veterinary services (PVS), which were universally neglected. Despite moderate cross-border collaboration in Clusters 1 to 4, Cluster 5’s scores indicate critical deficiencies that need immediate strategic attention. Analysis into compliance with International Health Regulations (IHR) further reflects the stark contrast between clusters, highlighting an urgent need for Cluster 5 to enhance its framework for adherence to international health standards.

The analysis of the risk environment unearthed paradoxical resilience patterns across clusters. While Cluster 5 exhibited high political-security stability (79.98) due to robust governance frameworks, it faced severe health system fragility. Conversely, Cluster 1 led in socioeconomic resilience (84.50), demonstrating the significant role of governance and infrastructure in shaping health security outcomes. The infrastructure adequacy in Cluster 5 (77.10), juxtaposed with its health system deficiencies, underscores the multifaceted nature of health security. ^58^

Those results stratified outcomes highlight the interdependence of governance, infrastructure, and specialized health capacities across clusters. The coexistence of robust infrastructure or political stability alongside health system fragility necessitates a nuanced approach to health security enhancement. ^4, 49^ Prioritizing the integration of dual-use research, developing medical countermeasures, and fostering cross-sectorial coordination emerge as paramount for strengthening the overall resilience of health security frameworks in non-EU countries. ^59^ By addressing these targeted interventions, nations can better prepare for and respond to future public health challenges, ultimately contributing to a more secure and healthy global community.

***4.3.2 Mapping Non-EU health security: Strengths, gaps, and policy implications across clusters***

The mapping of health security performance across non-EU countries reveals significant strengths and gaps, with clear policy implications for each cluster. High-performing nations in Cluster 1, comprising Armenia, Norway, and the United Kingdom, illustrate the efficacy of integrated governance frameworks. Their strengths are evident in several key areas, including public health-security authority coordination, effective infection control measures, robust compliance with International Health Regulations (IHR), transparency in surveillance practices, high immunization coverage, and effective antimicrobial resistance (AMR) management. Additionally, these countries demonstrate socioeconomic resilience and solid international commitments. Serving as exemplars of best practices, they provide a replicable blueprint for global health security enhancement that other nations could adopt. ^22^

However, even among these top performers, persistent gaps exist, particularly in dual-use research governance, emergency response planning, laboratory supply chains, zoonotic disease mitigation efforts, case-based investigation protocols, medical countermeasure deployment, and healthcare worker communication during crises. There is also a concerning lack of participation in Joint External Evaluations (JEE) and Performance of Veterinary Services (PVS), which indicates systemic vulnerabilities that require targeted interventions to bolster overall preparedness.

In contrast, Cluster 2, which includes nations like Albania, Georgia, Iceland, Israel, Russia, Switzerland, and Turkey, showcases moderate capabilities. Unfortunately, only 3% of their indicators exceed high-performance thresholds (>80), compared to 24% in Cluster 1. The persistent critical deficiencies, identified in 34% of practices—such as emergency response operations, real-time surveillance capabilities, healthcare worker communication, and dual-use research oversight—underscore the urgent need for comprehensive, multidimensional assessments that identify and address latent risks within these countries. ^59^

Cluster 3, labelled as “Intermediate,” features countries like Belarus, Kazakhstan, and Kyrgyz Republic, which exhibit polarized performance metrics. Approximately 29.7% of their practices achieve “good” levels (60–80), yet 54% fall below 40. Strengths in immunization rates, reporting under the IHR, and access to healthcare infrastructure are significantly overshadowed by critical weaknesses in key areas, including case-based investigations, medical countermeasures, and cross-sectorial coordination. This landscape necessitates urgent structural reforms aimed at elevating health security standards and addressing the identified gaps. ^47^

Cluster 4, referred to as “Warning,” encompasses nations such as Azerbaijan, Bosnia and Herzegovina, and Turkmenistan, representing a group facing systemic collapse in 70% of health security indicators. Severe deficiencies are noted particularly in biosecurity, surveillance systems, emergency preparedness, and medical countermeasure effectiveness, despite isolated strengths in immunization coverage. The vulnerabilities within these nations highlight the need for immediate international support to avert catastrophic health system failures during public health crises. ^60^

Lastly, Cluster 5, designated as “Dangerous,” includes Andorra, Monaco, San Marino, and Tajikistan. This group presents a paradox where competitive trade and travel restriction policies (e.g., scoring 90.63) coexist with critical failures across 70% of health security domains. Their disproportionately high political-security stability scores (79.98) obscure the underlying fragility of their health systems, signalling a pressing need for comprehensive governance overhauls. Aligning infrastructure investments with public health priorities is imperative to build a more resilient health security framework. ^61^

In addition, the disparities uncovered through this mapping exercise underscore the urgent need for targeted interventions across clusters to fortify health security. High-performing nations should continue to enhance their existing frameworks while sharing best practices to assist others. Meanwhile, lower-performing countries require international collaboration, increased funding, and capacity building to address specific weaknesses that impede their ability to effectively manage public health threats. By prioritizing these strategic interventions, non-EU nations can progress toward a more unified and effective global health security architecture.

## 5. Conclusion

This study, employing the Entropy-CoCoSo-K-means methodology, provides a comprehensive assessment of health security preparedness among non-EU countries, offering critical insights into regional prioritization patterns, performance rankings, and cluster-specific vulnerabilities. The findings reveal a complex landscape characterized by diverse levels of preparedness, varying priorities across health security dimensions, and significant disparities in capabilities across different country clusters.

The entropy-weighted analysis of health security indicators underscores the importance of strategic resource allocation, with a particular emphasis on strengthening detection and reporting mechanisms, scaling up preventive measures, and ensuring compliance with international norms. Cross-regional comparisons with the African Region and the Eastern Mediterranean Region (EMR) highlight the need for tailored strategies that address the unique challenges and priorities of each region.

The performance ranking of non-EU countries reveals a dynamic landscape, with some nations demonstrating improvements in their health security measures, while others experienced declines. The cluster analysis further elucidates the heterogeneity of health security preparedness, identifying distinct clusters of countries with varying levels of capabilities and vulnerabilities.

Strategic resource allocation must target universal weaknesses—such as laboratory supply chain resilience and zoonotic disease surveillance—while tailored interventions address distinct cluster profiles, from high-performing nations requiring dual-use research oversight to fragile systems needing urgent biosecurity support. The findings challenge homogenized approaches to health security, advocating instead for equity-driven metrics, institutionalized JEE/PVS assessments, and cross-cluster mentorship to redress systemic inequities in resource access and governance capacity. Future research must extend beyond the 2017–2021 scope to account for post-pandemic geopolitical shifts, validate findings through hybrid models (e.g., integrating neural networks), and expand comparisons to EU or global regions. By embedding these insights into policy, stakeholders can advance context-sensitive, multispectral strategies that strengthen health security resilience and foster equitable global health governance.

## Ethics and consent

No Ethical approval or consent needed.

## Declaration of generative AI and AI-assisted technologies in the writing process

During the preparation of this work the author(s) used [DeepSeek v3, Paperpal, Quillbot, and ChatGPT] for language refinement and structure. After using this tools, the author(s) reviewed and edited the content as needed and take(s) full responsibility for the content of the publication.

**Glossary**
CoCoSo (Combined Compromise Solution): A multi-criteria decision-making method that integrates multiple aggregation strategies to rank alternatives
EMR (Eastern Mediterranean Region): A World Health Organization (WHO) regional classification covering 22 countries in the Middle East, North Africa, and parts of Asia.
Entropy: Entropy Weight Approach
GHSI: Global Health Security Index – A comprehensive assessment of a country’s health security capabilities
HeS: Health security
MCDM: Multi-Criteria Decision-Making.
Non-EU countries: Non-EU countries in Europe refer to sovereign states geographically located within the European continent that are not members of the European Union (EU).

## Data Availability

The data supporting the findings of this study are publicly available and can be accessed through the following repository (Global Health Security Index, Global Health Security Index Data Model and Report. (2021), at https://ghsindex.org/report-model/). ^20^ Zenodo: MCDM-ML Based Tool for Health Security Optimization in Non-EU Countries Comprehensive Assessment and Visualization Workflow (V2.0.1) Doi: https://doi.org/10.5281/zenodo.15185666. ^38^ This project contains the following extended data:
• ProfAdelAbdulsalam-Supplementary-material--Software-de3938c • LICENSE • README.md • MCDM-ML Based Tool for Health Security Optimization in Non-EU Countries Comprehensive Assessment and Visualization Workflow.xlsm. All data, and processing results related to this study are presented in this file. This file integrates the processes of weighting, ranking, and clustering analyses into a single Excel-based tool, offering a comprehensive framework for analysis the Health Security inequalities in Non-EU European Countries:
- Health Security Indicator Compilation: Initiates data acquisition and selection, allowing users to access raw datasets for non-EU countries, African nations, and Eastern Mediterranean Region (EMR) regions across multiple years (2019, 2021, and 2017-2021 averages). - Entropy-Driven Criterion Weighting: Links to sub-menus for calculating objective weights of health security indicators, segmented by region (non-EU, EMR, Africa) and timeframe. - Entropy-CoCoSo Prioritization: Directs users to rank countries using the Combined Compromise Solution (CoCoSo) method, with options to analyse 2019, 2021, or averaged data. - K-Means Clustering: Facilitates grouping of countries into performance clusters based on health security scores. Source software available from: ( https://github.com/ProfAdelAbdulsalam/Supplementary-material--Software/blob/V2.0.1/MCDM-ML Based Tool for Health Security Optimization in Non-EU Countries Comprehensive Assessment and Visualization Workflow.xlsm) and archived via [ https://doi.org/10.5281/zenodo.15185666]. ^38^ License: OSI approved open license software is under CC0-1.0 license.
• The data and materials supporting the results and analyses of the paper.pdf This source includes the values behind the results reported in sections 3.1 and 3.2 of the paper. This file also includes the values behind the measures reported in all analysis and discussion sections, as well as the values used to construct tables and figures. The data supporting the findings of this study are publicly available and can be accessed through the following repository ( https://github.com/ProfAdelAbdulsalam/Supplementary-material--Software/blob/V2.0.1/ The data and materials supporting the results and analyses of the paper.pdf ) and archived via [ https://doi.org/10.5281/zenodo.15185666]. ^38^ This supplementary resource provides detailed support for replicating the study’s methods and results. Data are available under the terms of the CC0-1.0 license.
