# Supplementary material for: Health Security inequalities in Non-EU European Countries: A Cross-National Comparative Assessment Using an Integrated MCDM-Machine Learning Approach
Source: F1000Res. 2025 Aug 19;14:462. Originally published 2025 Apr 24. [Version 2] doi: 10.12688/f1000research.163662.2 (PMC12423625; doi:10.12688/f1000research.163662.2)
Supplement: Peer review history

# Reviewer report: Reviewer response for version 2

**DOI:** 10.5256/f1000research.186499.r406869

**Published:** 2025-09-11

**Authors:** Shaimaa Awadh Baha al_Deen

**Affiliations:** 1 Salahaddin University - Erbil, Erbil, Kurdistan Region, Iraq

Approved

 No comment

Is the work clearly and accurately presented and does it cite the current literature?

Yes

If applicable, is the statistical analysis and its interpretation appropriate?

Not applicable

Are all the source data underlying the results available to ensure full reproducibility?

Yes

Is the study design appropriate and is the work technically sound?

Yes

Are the conclusions drawn adequately supported by the results?

Yes

Are sufficient details of methods and analysis provided to allow replication by others?

Yes

Reviewer Expertise:

My research interests include Artificial Intelligence,  Machine Learning, Big Data, Data Science, Temporal Databases, Decision Support Systems, and Real Time Systems

I confirm that I have read this submission and believe that I have an appropriate level of expertise to confirm that it is of an acceptable scientific standard.

# Reviewer report: Reviewer response for version 2

**DOI:** 10.5256/f1000research.186499.r406868

**Published:** 2025-09-10

**Authors:** Mohammed S. Abdo

**Affiliations:** 1 Hodeidah University, Al-Hudaydah, Yemen

The revised version submitted by the authors has adequately addressed the earlier comments and is acceptable in its current form.

Is the work clearly and accurately presented and does it cite the current literature?

Yes

If applicable, is the statistical analysis and its interpretation appropriate?

Not applicable

Are all the source data underlying the results available to ensure full reproducibility?

Yes

Is the study design appropriate and is the work technically sound?

Partly

Are the conclusions drawn adequately supported by the results?

Yes

Are sufficient details of methods and analysis provided to allow replication by others?

Yes

Reviewer Expertise:

Mathematical Models, Nonlinear Dynamics

I confirm that I have read this submission and believe that I have an appropriate level of expertise to confirm that it is of an acceptable scientific standard.

# Reviewer report: Reviewer response for version 1

**DOI:** 10.5256/f1000research.180050.r381103

**Published:** 2025-08-05

**Authors:** Mohammed S. Abdo

**Affiliations:** 1 Hodeidah University, Al-Hudaydah, Yemen

This article tackles an important topic using a creative and technically capable methodology. However, to meet the standards of scientific rigor and transparency, it must address methodological clarity, provide deeper interpretation, and engage with the relevant literature. With these enhancements, the article would significantly contribute to comparative public health policy and health security research.

 e.g.,

 1) Clarity: Define all six indicators and aggregation methods explicitly.

 2) Methodology: Justify methodological choices and validate clustering results statistically.

 3) Interpretation: Deepen analysis of surprising findings (e.g., low “risk environment” weight).

 4) Literature: Add references to similar HeS comparative studies.

 5) Policy Implications: Offer specific, actionable interventions for low-performing countries.

 6) Reproducibility: Provide a flowchart or pseudocode for the full analytical pipeline.

 Recommendation: Minor Revision

Is the work clearly and accurately presented and does it cite the current literature?

Yes

If applicable, is the statistical analysis and its interpretation appropriate?

Not applicable

Are all the source data underlying the results available to ensure full reproducibility?

Yes

Is the study design appropriate and is the work technically sound?

Partly

Are the conclusions drawn adequately supported by the results?

Yes

Are sufficient details of methods and analysis provided to allow replication by others?

Yes

Reviewer Expertise:

Mathematical Models, Nonlinear Dynamics

I confirm that I have read this submission and believe that I have an appropriate level of expertise to confirm that it is of an acceptable scientific standard, however I have significant reservations, as outlined above.

# Reviewer report: Reviewer response for version 1

**DOI:** 10.5256/f1000research.180050.r381101

**Published:** 2025-05-21

**Authors:** Shaimaa Awadh Baha al_Deen

**Affiliations:** 1 Salahaddin University - Erbil, Erbil, Kurdistan Region, Iraq

Prof. Shaimaa Awadh Baha al-Deen, Salahaddin University, Kurdistan Region, Erbil, Iraq

 Title of Paper: Health Security Inequalities in Non-EU European Countries: A Cross-National Comparative Assessment Using an Integrated MCDM-Machine Learning Approach

 Authors: Mijahed Nasser Aljober, Adel A. Nasser, Abed Saif Ahmed, Alghawli et al

 This study presents a framework for assessing health security performance and prioritization in non-EU countries in Europe by analyzing data from the Global Health Security Index (GHSI) for 2019 and 2021. The key questions addressed are critical for policymakers: (1) What is the current status of health security in these countries? (2) How are health security indicators prioritized now, and what changes occurred between 2019 and 2021? (3) Which aspects of health security should Non-EU countries focus on or invest in to enhance their health security? The authors utilize recognized quantitative methods to answer these questions.

 The findings of this paper are especially significant for policymakers and stakeholders in the region, highlighting the importance of comprehensive and ongoing strategies for health security. In summary, the study adds important knowledge to the field, advocating for a transition to stronger and more integrated health systems to enhance preparedness for future challenges.

 Here are some recommendations and observations for enhancing the manuscript:

 Introduction Section: What scientific contribution does your paper make to the field? Can you clarify the scientific implications of your work?

 Methods: The authors should elaborate on the GHSI data, specifically whether they used the original 2019 dataset for their data and rankings or the revised 2019 dataset released alongside the 2021 GHSI, as there are minor differences that could impact the study’s findings.

 Discussion: 1) On page 16, the term “Global Health Security Index” should replace "Health Security Index." 2) For the clustering results in Table 4, it would be beneficial to include a graphical representation in two-dimensional space, with clustering algorithms showing the groupings of countries for better visualization. 3) The discussion section contains valuable insights; could you relate these to any existing non-EU health programs?

 Conclusions: The Conclusion section could be expanded to discuss the limitations of this study and suggest future research directions.

 I recommend indexing the paper with minor revisions to address the comments outlined above (Approved).

 I confirm that I have read this submission and believe that I have an appropriate level of expertise to confirm that it is of an acceptable scientific standard.

Is the work clearly and accurately presented and does it cite the current literature?

Yes

If applicable, is the statistical analysis and its interpretation appropriate?

Not applicable

Are all the source data underlying the results available to ensure full reproducibility?

Yes

Is the study design appropriate and is the work technically sound?

Yes

Are the conclusions drawn adequately supported by the results?

Yes

Are sufficient details of methods and analysis provided to allow replication by others?

Yes

Reviewer Expertise:

My research interests include Artificial Intelligence,  Machine Learning, Big Data, Data Science, Temporal Databases, Decision Support Systems, and Real Time Systems

I confirm that I have read this submission and believe that I have an appropriate level of expertise to confirm that it is of an acceptable scientific standard.

# Response

**Published:** 2025-08-11

**Authors:** Adel A. Nasser

**Affiliations:** Information Systems, Not Applicable, Sa'ada, Sa'ada, Yemen

**Dear Prof. Shaimaa Awadh Baha al_Deen,**

 Thank you for your thoughtful review and for the insightful comments provided on our manuscript. Your feedback has been invaluable in guiding our revisions.

**Comment 1:** Introduction Section: What scientific contribution does your paper make to the field? Can you clarify the scientific implications of your work?

**Response:** Thank you for your valuable feedback , we have revised the Introduction to more explicitly state the scientific contribution of our work, emphasizing its role in providing a novel, multidimensional framework for assessing health security in a previously under-researched cohort of countries.

**Comment 2:** Methods: The authors should elaborate on the GHSI data, specifically whether they used the original 2019 dataset for their data and rankings or the revised 2019 dataset released alongside the 2021 GHSI, as there are minor differences that could impact the study’s findings.

**Response:** Thank you for your valuable feedback regarding this section. We have clarified in the Methods section that our analysis utilizes the revised 2019 GHSI dataset that was released alongside the 2021 report to ensure consistency and comparability across timeframes

**Comment 3**: Discussion: 1) On page 16, the term “Global Health Security Index” should replace "Health Security Index." 2) For the clustering results in Table 4, it would be beneficial to include a graphical representation in two-dimensional space, with clustering algorithms showing the groupings of countries for better visualization. 3) The discussion section contains valuable insights; could you relate these to any existing non-EU health programs?

**Response:** Thank you for your insightful comment. As suggested, we have corrected the term "Health Security Index" to "Global Health Security Index" on page 16. We also appreciate the suggestion to include a graphical representation of the clustering results. We have added a radar chart plot (Figure 5) that visually depicts the country groupings, making the cluster analysis more intuitive. We have also expanded the Discussion section to connect our findings to existing non-EU health initiatives and frameworks, such as the WHO's European Programme of Work, to better contextualize our policy recommendations.

**Comment 4:** Conclusions: The Conclusion section could be expanded to discuss the limitations of this study and suggest future research directions

**Response:** Thank you for your insightful comment regarding the inclusion of a limitations and future work section. In response, the conclusion section has been expanded to include a dedicated paragraph on the study's limitations and to suggest specific directions for future research, including the integration of qualitative data and the use of more advanced machine learning models.

 We hope that these responses adequately address your concerns and further improve our manuscript. Thank you again for your valuable insights!
